# Supplementary material for: Large area polymer semiconductor sub-microwire arrays by coaxial focused electrohydrodynamic jet printing for high-performance OFETs
Source: Nat Commun. 2022 Oct 20;13:6214. doi: 10.1038/s41467-022-34015-z (PMC9584972; doi:10.1038/s41467-022-34015-z)
Supplement: Supplementary file 1 — Supplementary Information [file 41467_2022_34015_MOESM1_ESM.pdf]

Supplementary Information

Large area polymer semiconductor sub-microwire arrays by coaxial focused electrohydrodynamic jet printing for high-performance OFETs

Wang et.al

Supplementary Figures

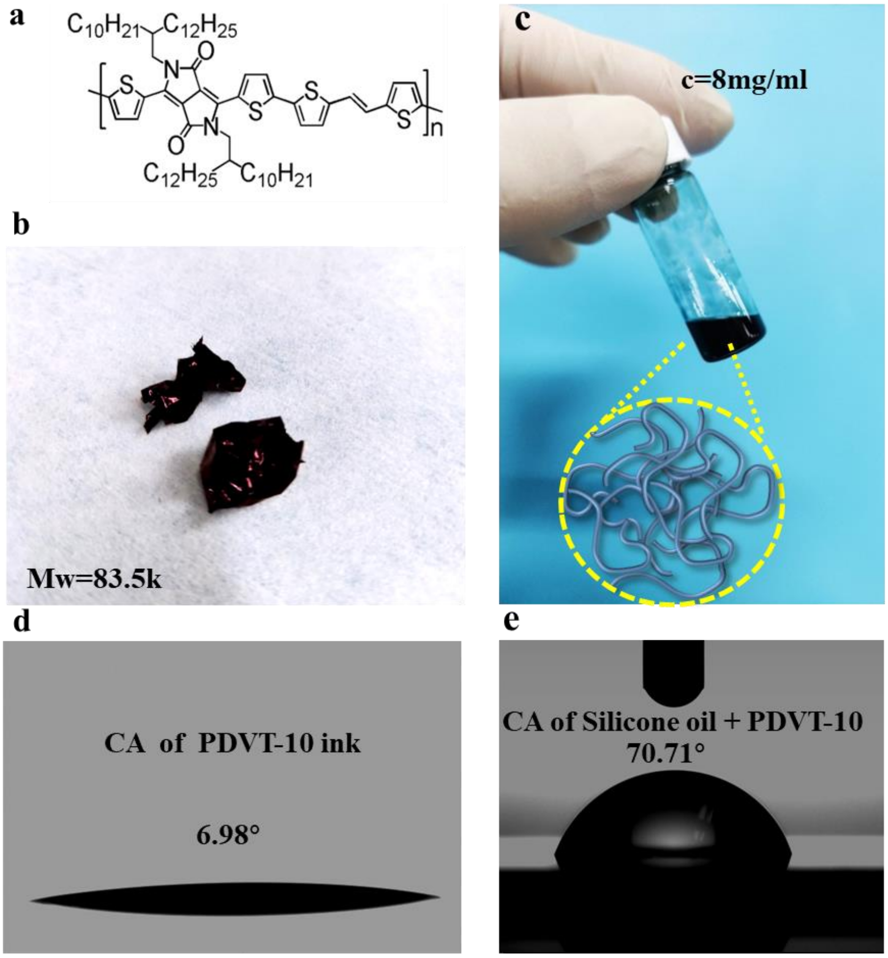

Supplementary Fig. 1 Properties of polymer semiconductor PDVT-10. a Molecular structure of PDVT-10. **b** The solid of PDVT-10. **c** Polymer semiconductor solution and the schematic diagram of random arrangement of polymer fibers in the solution. **d, e** Contact angle of PDVT-10 ink and Silicone oil/PDVT-10 double layer liquid.

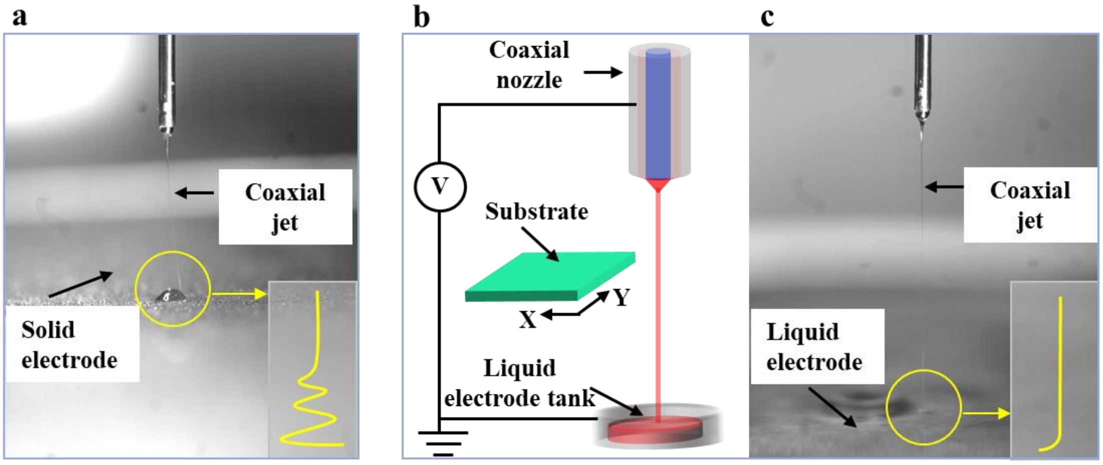

Supplementary Fig. 2. a Image of CFEJ printing process with solid electrode. **b** Schematic of liquid electrode for CFEJ printing. **c** Image of CFEJ printing process with liquid electrode.

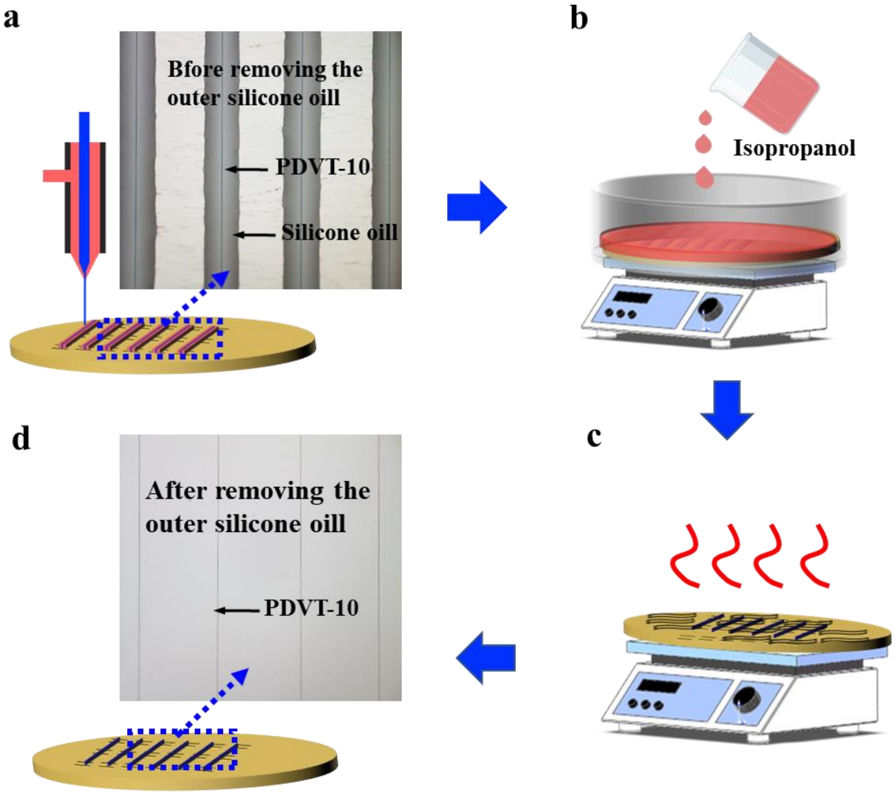

Supplementary Fig. 3. Flow chart of removing outer high viscosity liquid by solution method. **a** The printed wrapped structures. **b** Removing the outer layer solution of silicone oil in isopropanol at 70 ℃. **c** Evaporation of residual solvents and annealing polymer at a temperature of 180 ℃. **d** The inner structure obtained after removing the outer layer solution.

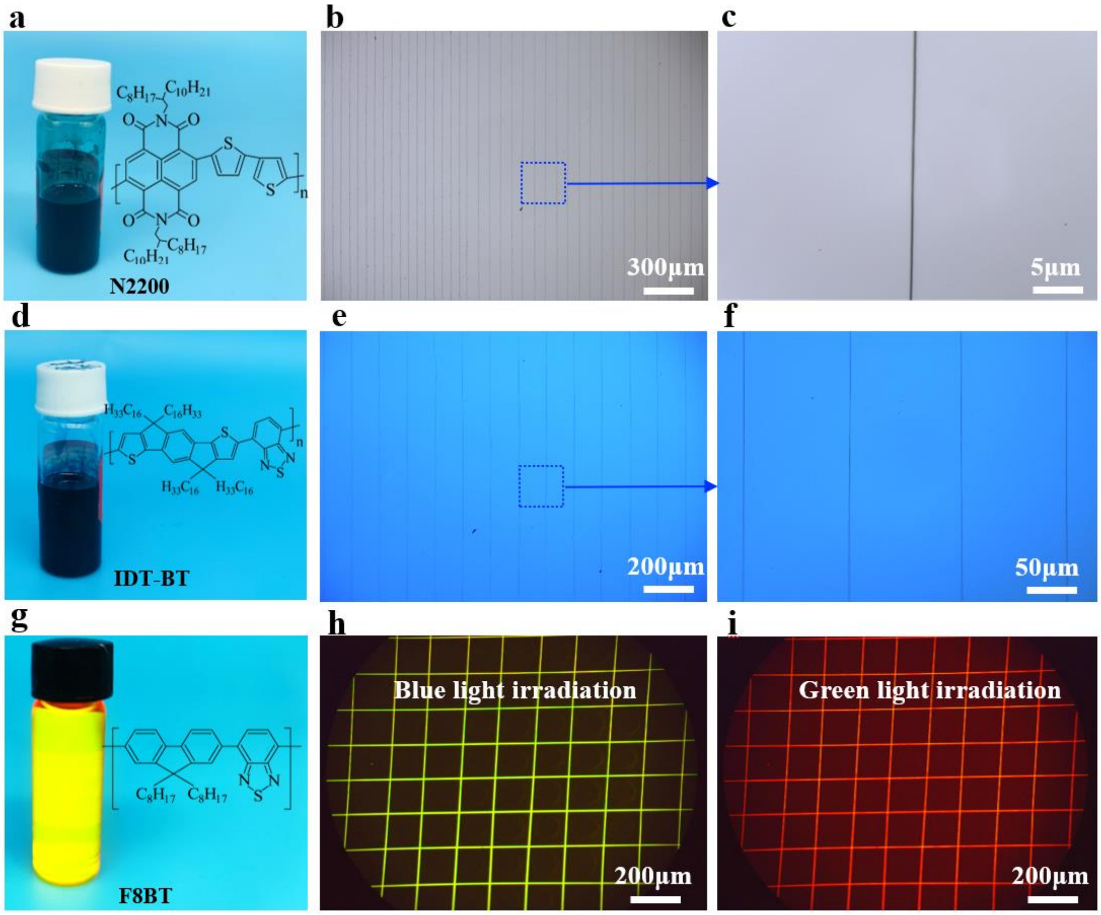

Supplementary Fig. 4. a-c Linear array structures of N2200 polymer. **d-f** Linear array structures of IDT-BT polymer. **g-i** Crossing structures of F8BT polymer.

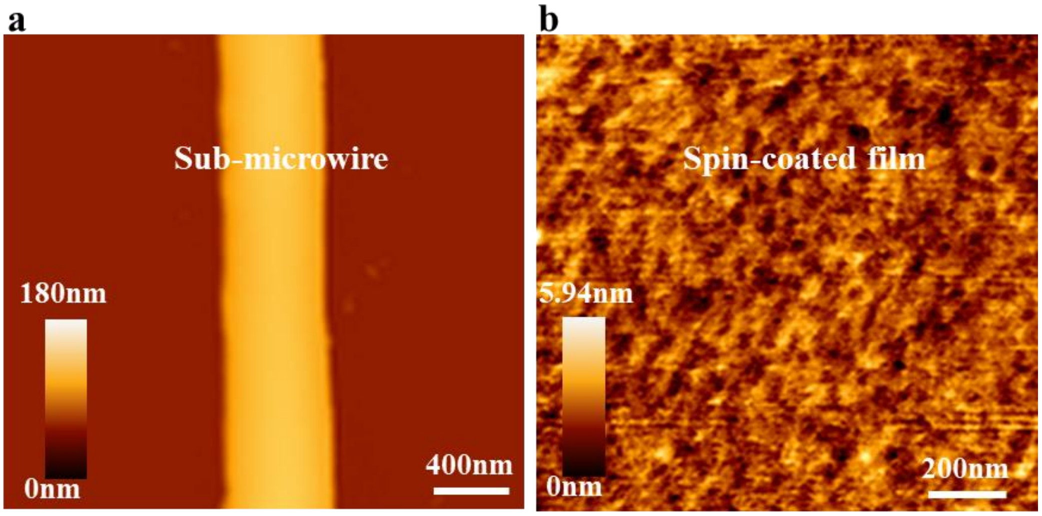

Supplementary Fig. 5. AFM image of sub-microwire and spin-coated thin film. **a** AFM of single PDVT-10 sub-microwire. **b** AFM of spin-coated PDVT-10 thin film.

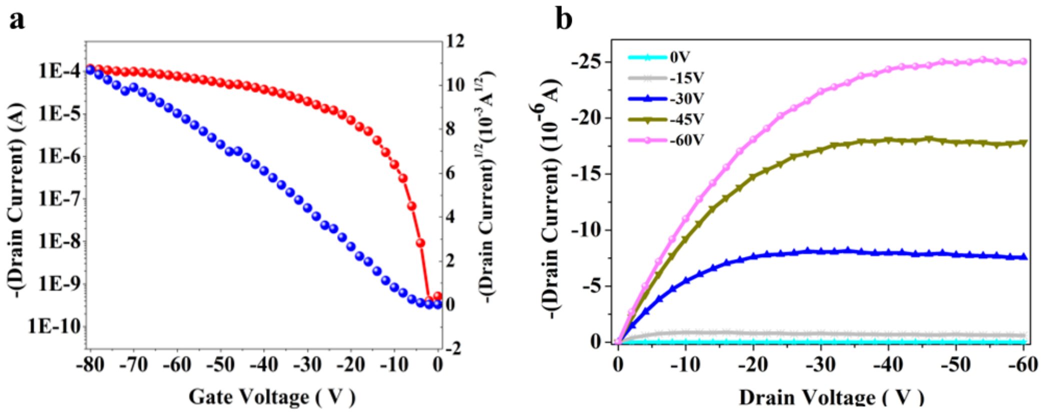

Supplementary Fig. 6. a Transfer characteristic of the thin film based OFETs. **b** Output characteristic of the thin film based OFETs.
