# Supplementary material for: Large area polymer semiconductor sub-microwire arrays by coaxial focused electrohydrodynamic jet printing for high-performance OFETs
Source: Nat Commun. 2022 Oct 20;13:6214. doi: 10.1038/s41467-022-34015-z (PMC9584972; doi:10.1038/s41467-022-34015-z)
Supplement: Supplementary file 3 — Description of Additional Supplementary Files [file 41467_2022_34015_MOESM3_ESM.pdf]

File name: Supplementary Movie 1

Description: The printing process of coaxial focused electrohydrodynamic jet (CFEJ).
